# Supplementary material for: Fluid shear stress activates YAP1 to promote cancer cell motility
Source: Nat Commun. 2017 Jan 18;8:14122. doi: 10.1038/ncomms14122 (PMC5253685; doi:10.1038/ncomms14122)

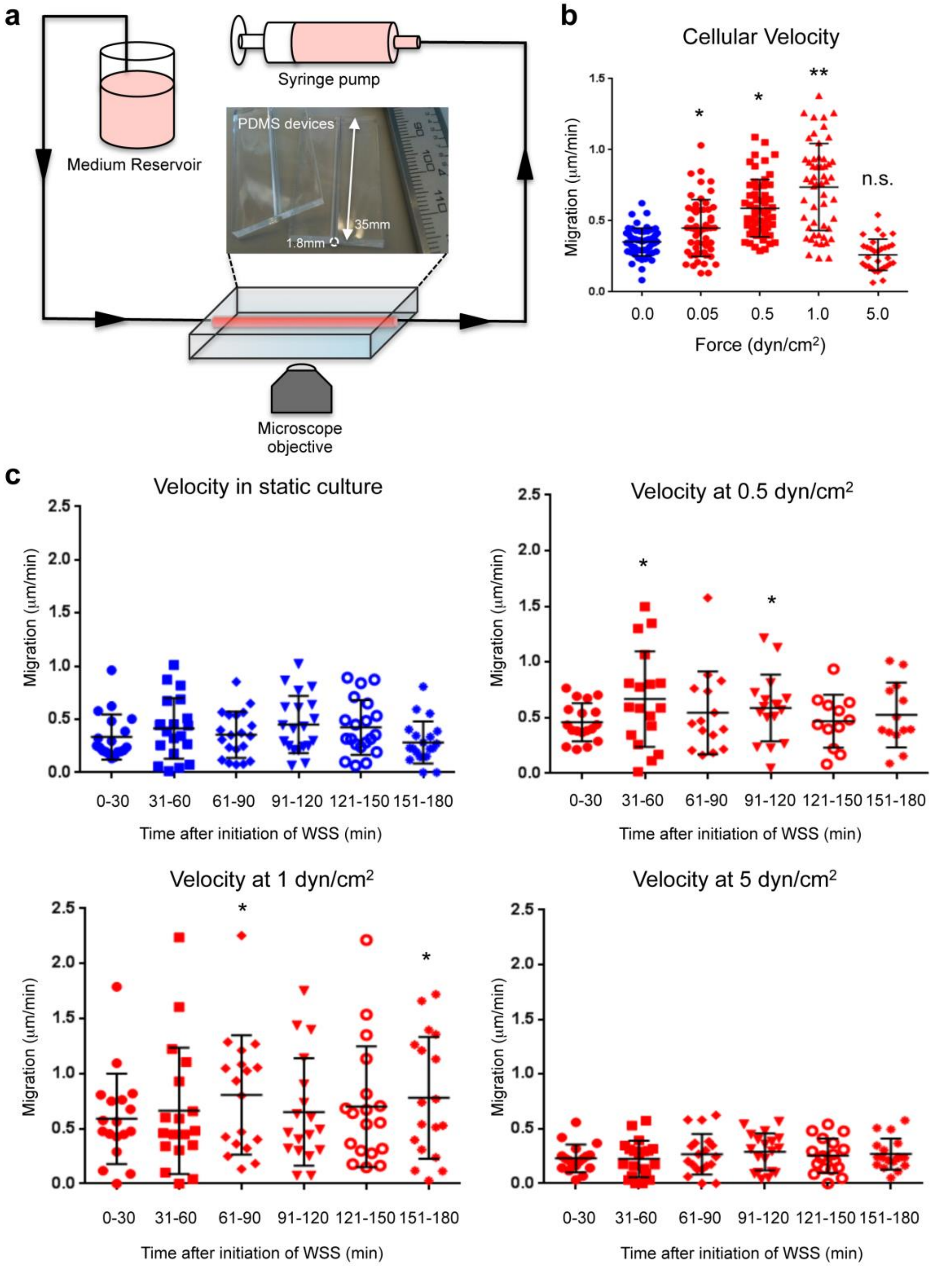
a
Medium Reservoir
Syringe pump
PDMS devices
35mm
1.8mm
Microscope objective
b
Cellular Velocity
Migration (μm/min)
0.0 0.05 0.5 1.0 5.0
Force (dyn/cm2)
n.s.
c
Velocity in static culture
Velocity at 0.5 dyn/cm2
Velocity at 1 dyn/cm2
Velocity at 5 dyn/cm2
0-30 31-60 61-90 91-120 121-150 151-180
Time after initiation of WSS (min)

Supplementary Figure 1. Biomimetic platform for application of fluid flow permits assessment of morphological response to WSS under compliant conditions

(*a*) PDMS polymer was cast to create a cylindrical culture surface by soft lithography. Following preincubation of the channel with collagen, prostate cancer cells were seeded uniformly by rolling across the culture surface with a vertical rotator. Fluidics driven by a syringe pump controlled WSS intensity. (*b*) Motility response of PC3 to WSS ranging from 0.05 to 5 dyn/cm2 compared in parallel reveals a finite range of magnitudes that stimulate cell movement on soft polymer (Kruskal-Wallis One-way ANOVA, *$P < 0.05$, **$P < 0.01$). (*c*) Latency to response decreases as intensity of WSS increases (Kruskal-Wallis One-way ANOVA, statistical comparisons are relative to static, *$P < 0.05$). Error bars represent ± SD.

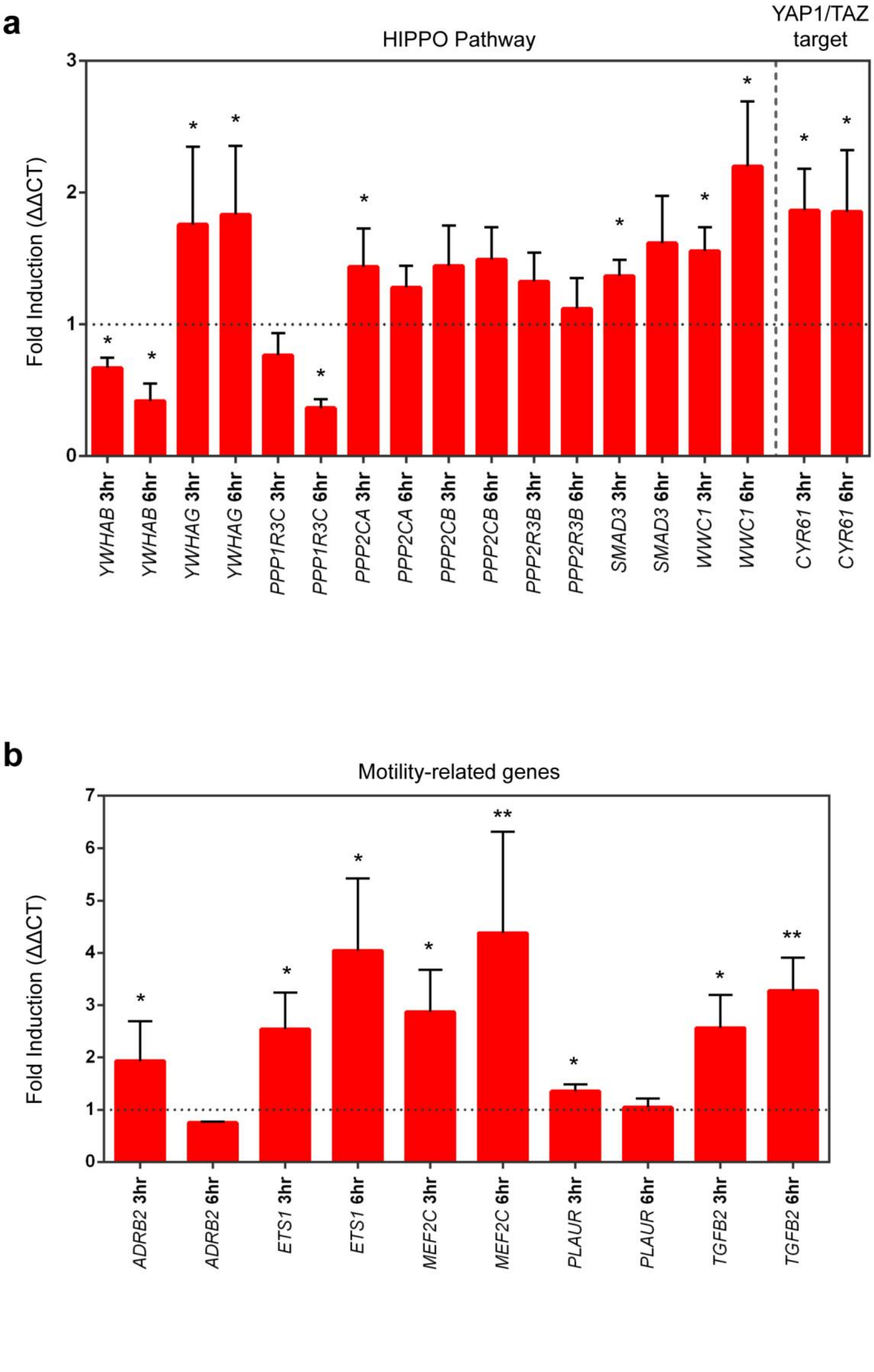
a
HIPPO Pathway
YAP1/TAZ target
Fold Induction (ΔΔCT)
3
2
1
0
YWHAB 3hr
YWHAB 6hr
YWHAG 3hr
YWHAG 6hr
PPP1R3C 3hr
PPP1R3C 6hr
PPP2CA 3hr
PPP2CA 6hr
PPP2CB 3hr
PPP2CB 6hr
PPP2R3B 3hr
PPP2R3B 6hr
SMAD3 3hr
SMAD3 6hr
WWC1 3hr
WWC1 6hr
CYR61 3hr
CYR61 6hr
b
Motility-related genes
Fold Induction (ΔΔCT)
7
6
5
4
3
2
1
0
ADRB2 3hr
ADRB2 6hr
ETS1 3hr
ETS1 6hr
MEF2C 3hr
MEF2C 6hr
PLAUR 3hr
PLAUR 6hr
TGFB2 3hr
TGFB2 6hr

Supplementary Figure 2. Validation of gene expression changes induced by WSS

Expression was validated by qRT-PCR of genes involved in (*a*) the HIPPO pathway and (*b*) motility, using samples prepared independently of those subjected to analysis by Illumina BeadChips. Error bars represent ± SEM.

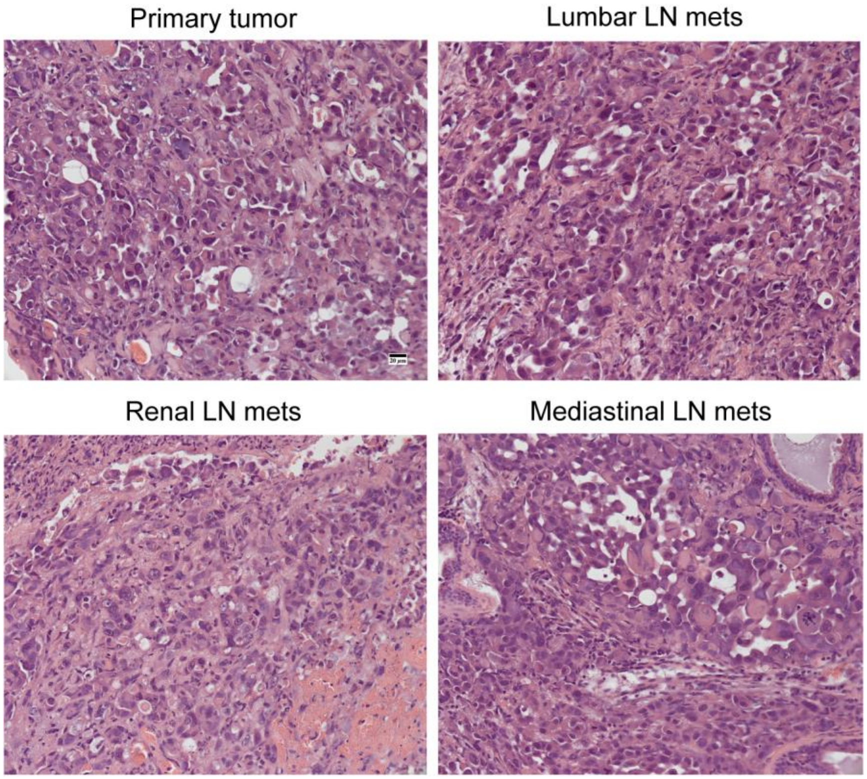

Supplementary Figure 3. H&E staining of primary tumors and lymph nodes

H&E staining images in primary tumor and lymph node metastases (LN mets) of representative mouse implanted orthotopically with PC3 cells. Scale bar = 20 μm.

a

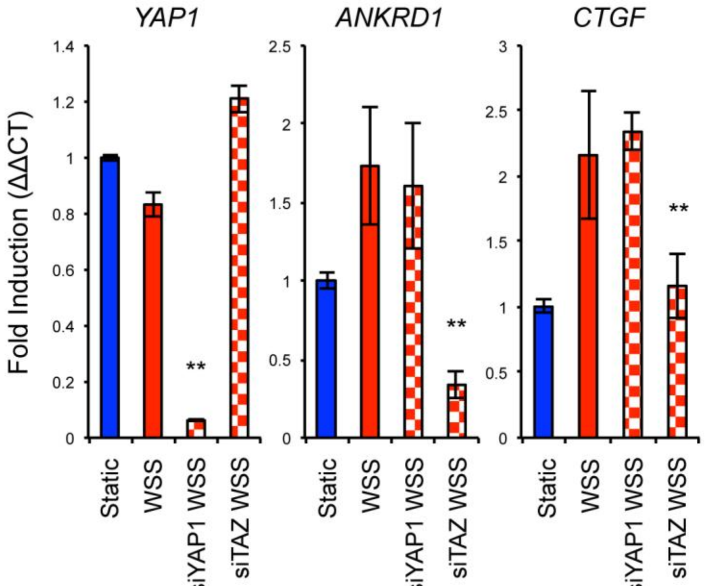
YAP1
ANKRD1
CTGF
Fold Induction (ΔΔCT)
1.4
1.2
1
0.8
0.6
0.4
0.2
0
2.5
2
1.5
0.5
3
**
Static
WSS
siYAP1 WSS
siTAZ WSS

b

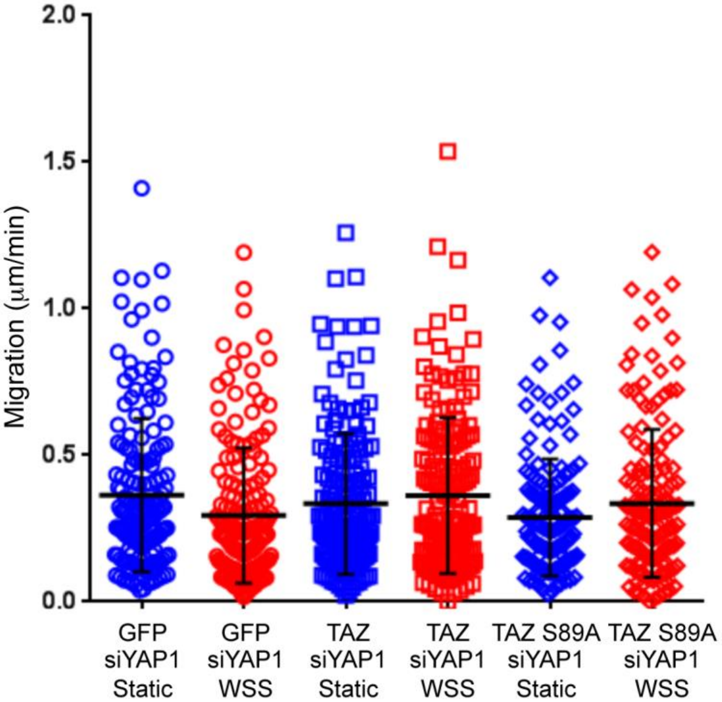
Migration (μm/min)
2.0
1.5
1.0
0.5
0.0
GFP siYAP1 Static
GFP siYAP1 WSS
TAZ siYAP1 Static
TAZ siYAP1 WSS
TAZ S89A siYAP1 Static
TAZ S89A siYAP1 WSS

Supplementary Figure 4. TAZ does not contribute to cell motility, but does regulate *ANKRD1* and *CTGF* in response to WSS

(*a*) Plasmids driving expression of EGFP, wild-type TAZ, or TAZ S89A were transfected into PC3 cells along with YAP siRNA. Migration speed was not enhanced by WSS in cells expressing wild-type or constitutively active TAZ (n=3 independent experiments, Kruskal-Wallis One-way ANOVA, not significant). (*b*) *TAZ* siRNA knockdown reduces *ANKRD1* and *CTGF* transcripts but *YAP1* appears to be dispensable for their upregulation by WSS (n=3 independent experiments, One-way ANOVA, **$P < 0.0001$). Error bars represent ± SEM.

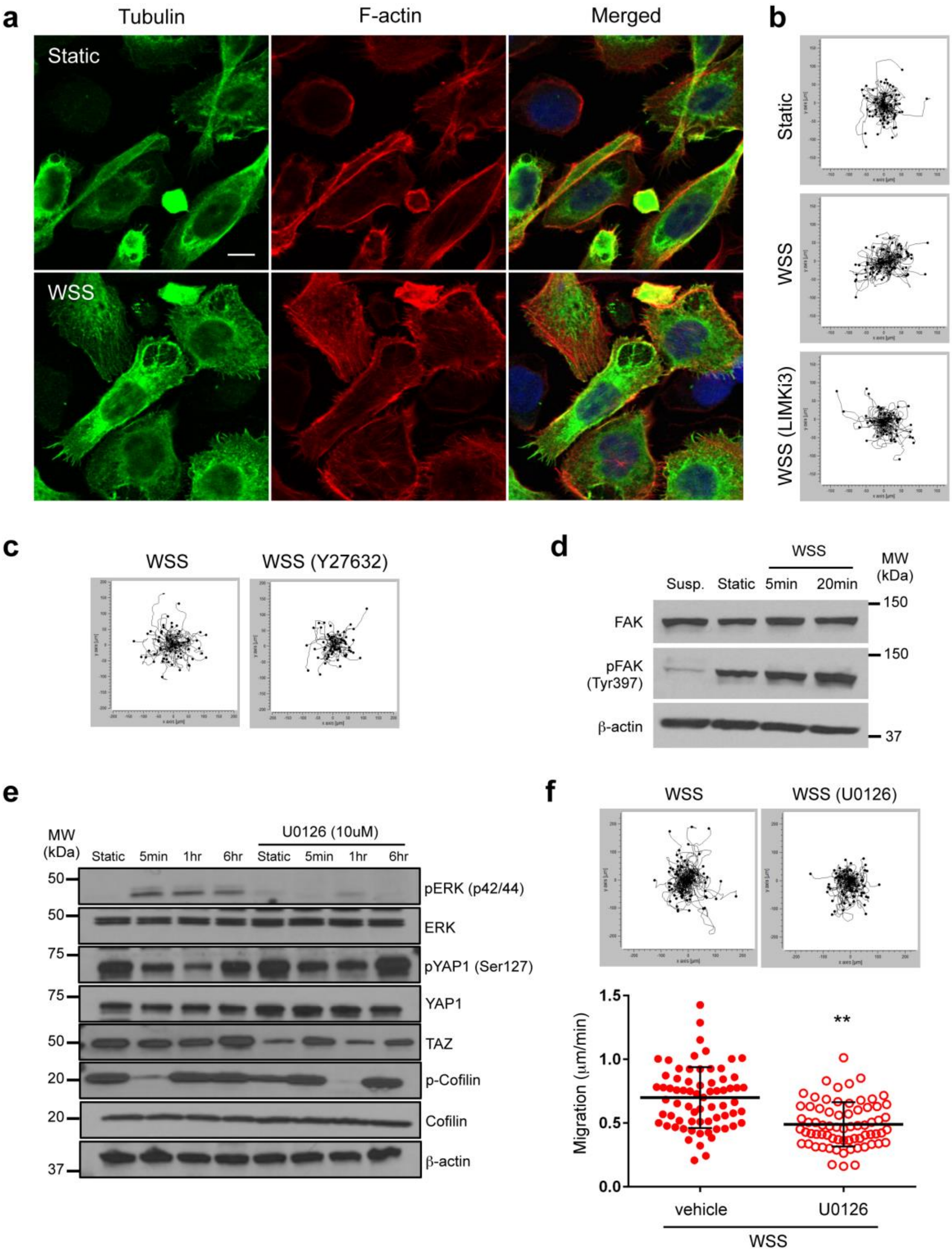

a
Tubulin
F-actin
Merged
Static
WSS
b
Static
WSS
WSS (LIMKi3)
c
WSS
WSS (Y27632)
d
WSS
MW (kDa)
Susp.
Static
5min
20min
FAK
pFAK (Tyr397)
β-actin
150
150
37
e
MW (kDa)
U0126 (10uM)
Static
5min
1hr
6hr
Static
5min
1hr
6hr
pERK (p42/44)
ERK
pYAP1 (Ser127)
YAP1
TAZ
p-Cofilin
Cofilin
β-actin
50
50
75
75
50
20
20
37
f
WSS
WSS (U0126)
Migration (μm/min)
1.5
1.0
0.5
0.0
**
vehicle
U0126
WSS

Supplementary Figure 5. WSS enhanced motility requires signaling via ROCK-LIMK and ERK

(*a*) PC3 cells were stained with tubulin and phalloidin F-actin after WSS exposure for 3 hr. Nuclei were detected by Draq5. Scale bar is equivalent to 10 μm. (*b*) Plots depict migratory paths of PC3 cells cultured with or without LIMKi3. (*c*) Inhibition of ROCK by Y27632 blocks WSS-induced cell migration. (*d*) Static cultured PC3 cells on collagen-coated tissue culture plastic without PDMS express high levels of pFAK Y397; whereas, cells in suspension express very low levels of pFAK Y397. WSS elevates FAK activity via increased phosphorylation of Y397 on these rigid surfaces. (*e,f*) U0126 MEK inhibitor interrupts ERK activation and migration and has minor effects on YAP1 dephosphorylation (Unpaired *t*-test, ***P* < 0.001). Error bars represent ± SD.

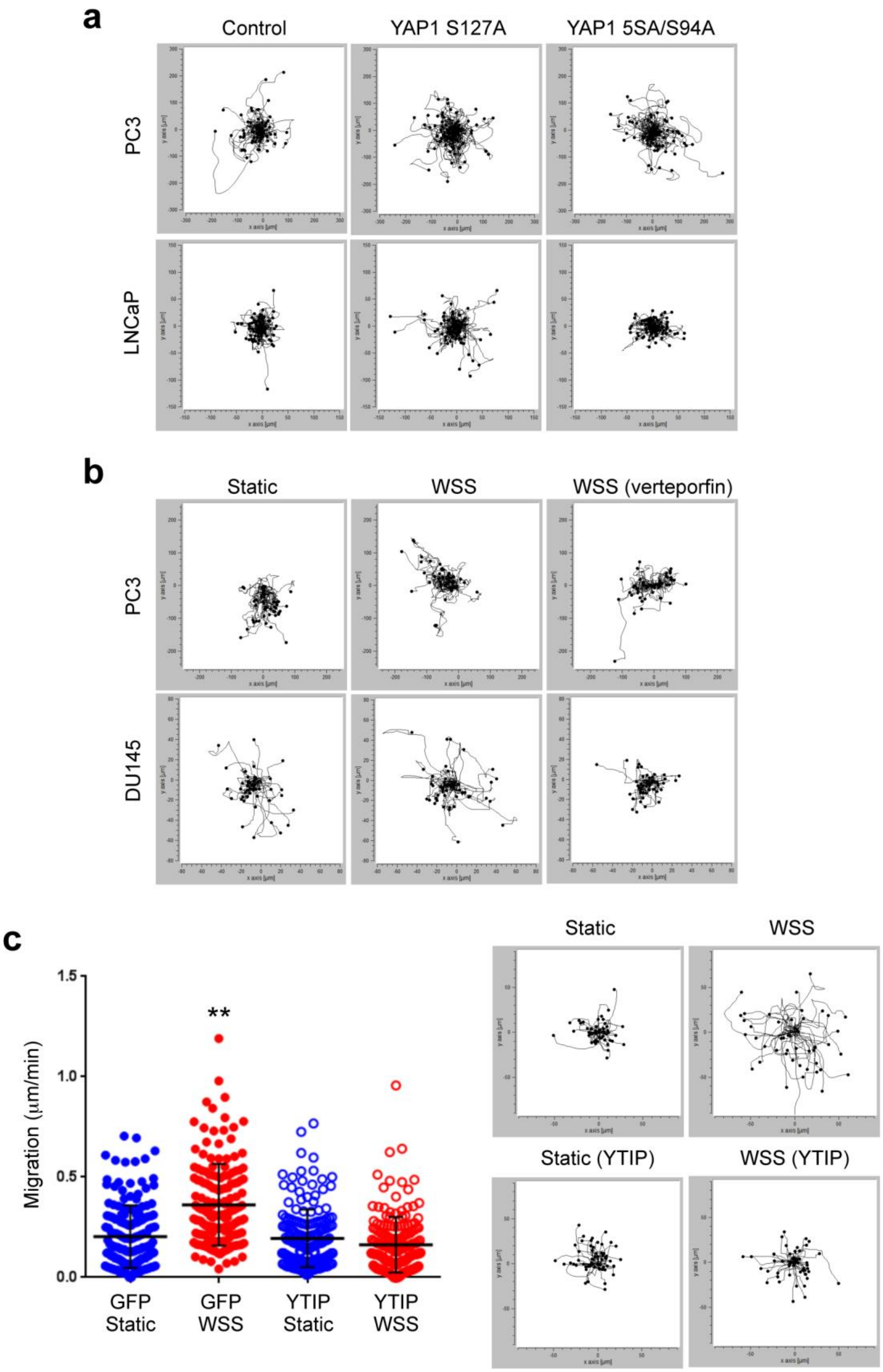
a
Control
YAP1 S127A
YAP1 5SA/S94A
PC3
LNCaP
b
Static
WSS
WSS (verteporfin)
PC3
DU145
c
Migration (μm/min)
**
GFP Static
GFP WSS
YTIP Static
YTIP WSS
Static
WSS
Static (YTIP)
WSS (YTIP)

Supplementary Figure 6. Intact YAP1-TEAD binding function is required for enhanced cell migration downstream of WSS

(a) Representative plots of cell migration following transfection with pEGFP-N1, YAP1 S127A or YAP1 5SA/S94A in PC3 or LNCap cell lines. *(b)* Motility plots of PC3 and DU145 cell lines depict dramatic reduction in cellular velocity when cells are treated with a small molecule, verteporfin that interrupts the YAP1-TEAD interaction. (*c*) Migration speed of YTIP (YAP-TEAD inhibitory peptide)-transfected cells is significantly reduced in WSS (n=3 independent experiments, Kruskal-Wallis One-way ANOVA, **P<0.001). Plots show inhibition of motility in cells expressing YTIP. Error bars represent ± SEM.

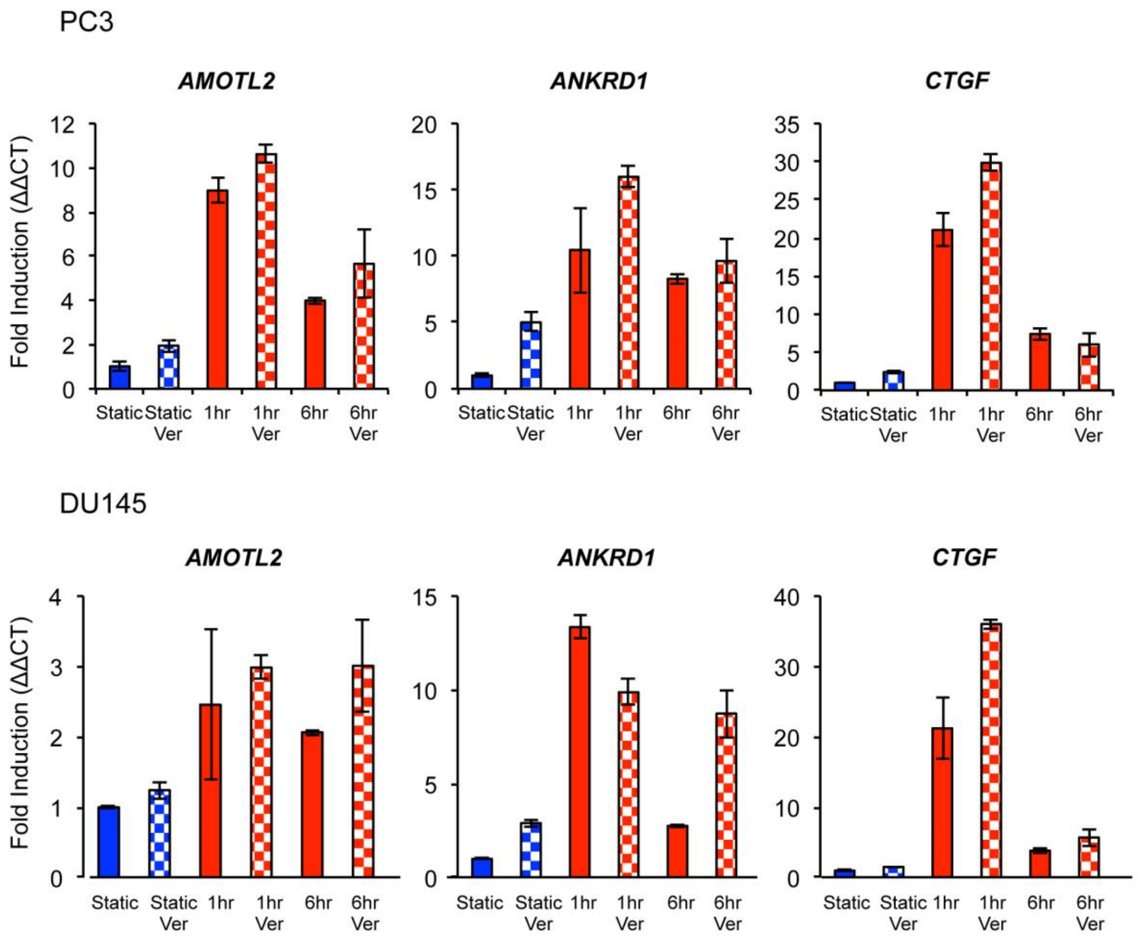

Supplementary Figure 7. Verteporfin treatment reveals YAP1-TEAD independent induction of YAP1/TAZ target genes by WSS

Expression analysis of YAP1/TAZ target genes by qRT-PCR points to WSS induction independent of YAP1-TEAD interaction. Ver, verteporfin. Error bars represent ± SEM.

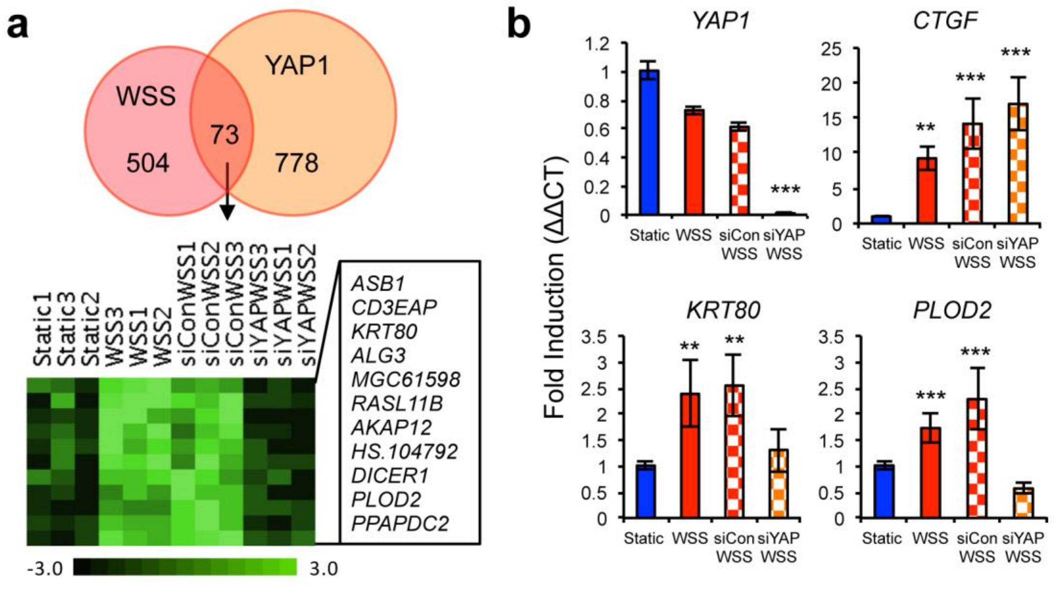

Supplementary Figure 8. YAP1 modulates effectors of cell motility, proliferation, and survival downstream of WSS

(*a*) A subset of genes altered by WSS are regulated by YAP1, as determined by comparisons between static and WSS, and control siRNA and *YAP1* siRNA treated cells exposed to WSS. 504 genes are regulated by WSS independently of *YAP1* and 73 genes require *YAP1*. Heatmap depicts 11 shared genes which are upregulated by WSS and downregulated by *YAP1* knockdown ($P < 0.05$, 2-fold threshold). (*b*) Validation of gene expression by qRT-PCR (One-way ANOVA, **$P < 0.01$, ***P<0.0001 and ***$P < 0.0001$ for *YAP1* and *CTGF*, **$P = 0.0092$ for *KRT80*, ***$P = 0.0004$ for *PLOD2*). Error bars represent ± SEM.

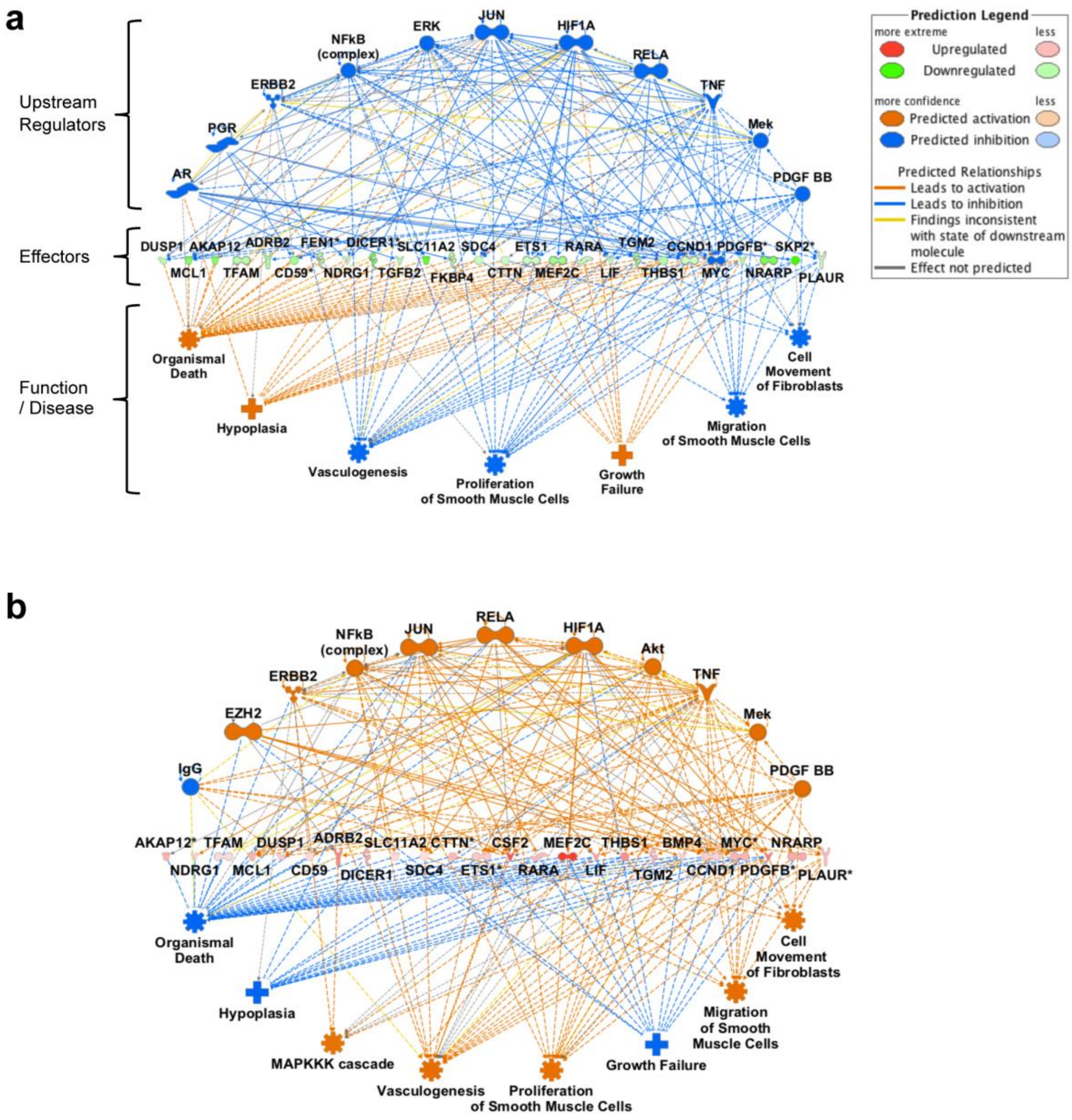

Supplementary Figure 9. Network of processes regulated by WSS

(*a*) Network of processes regulated by WSS-induced YAP1 signaling shows that YAP1 acts through master regulators to drive motility, proliferation, and survival, and these diseases/functions are negatively impacted by *YAP1* knockdown (*P* < 0.05, 1.25-fold threshold for genes changed in siCon-siYAP1 comparison). (*b*) WSS modulates master regulators to drive cell migration, proliferation, and survival aided by YAP1 (*P* < 0.05, 1.25-fold threshold for genes changed in static vs WSS).

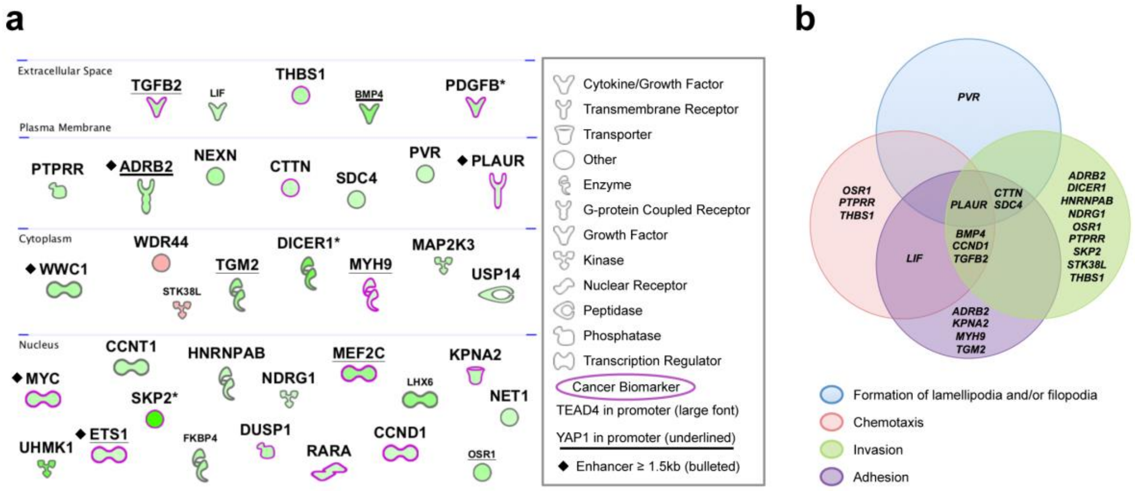

Supplementary Figure 10. Proteins encoded by 36 YAP1-regulated genes known to affect cell migration

(*a*) Gene promoters were evaluated *in silico* for evidence of YAP1/TEAD4 binding in ChIP-seq data (large font size depicts TEAD4 binding identified in ENCODE annotations; underlined font depicts YAP1 binding found by Lin et al. and Stein et al.[23,24]; bullet mark indicates genes regulated by enhancers in Zanconato et al.[25]). Internal shading within molecules represents expression of siYAP-treated cells relative to siCon under WSS conditions (log ratio of intensity values; green represents downregulation and red depicts upregulation with siYAP treatment). Magenta edge around molecules indicates that the highlighted gene is a cancer biomarker linked to diagnosis and/or prognosis.

(b) Venn diagram shows unique and overlapping metastatic functions of 36 genes. Blue corresponds with roles in formation of lamellipodia and filopodia, pink with chemotaxis, green with invasion, and purple with regulation of adhesion.

Supplementary Figure 11. Full gel blots

Figure 3c

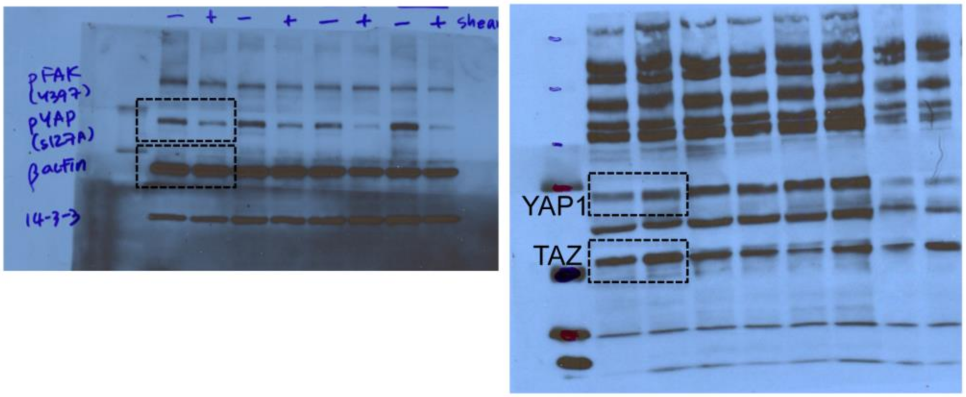

Figure 3e

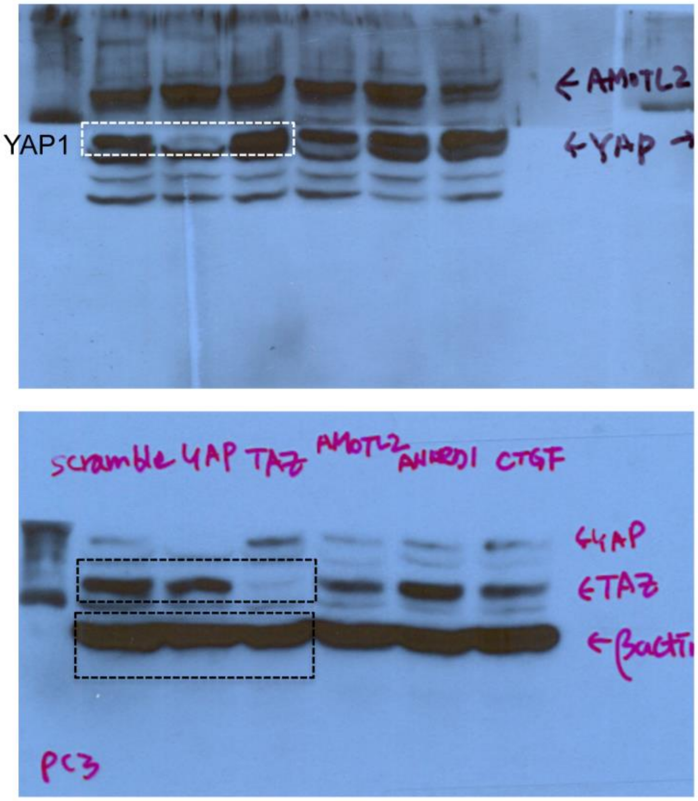

Supplementary Figure 11. Full gel blots (continued)

Figure 4a

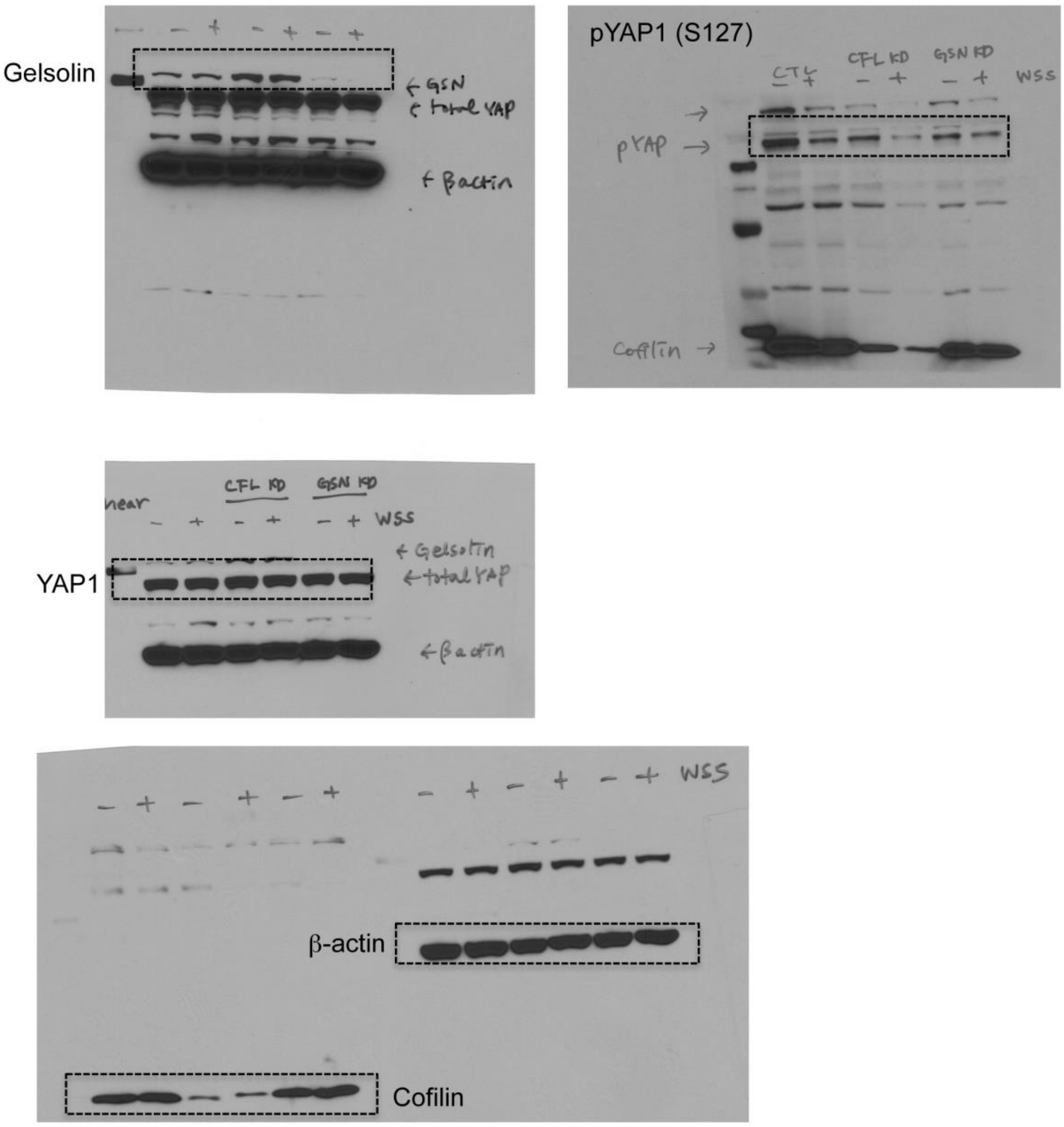

Supplementary Figure 11. Full gel blots (continued)

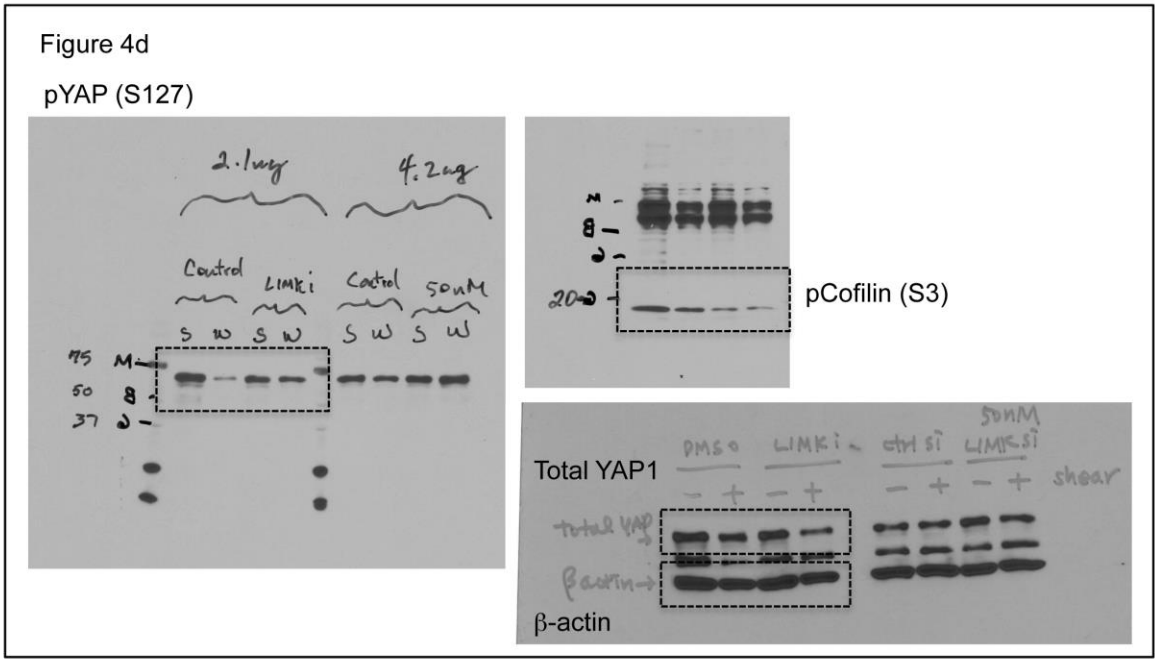

Supplementary Figure 11. Full gel blots (continued)

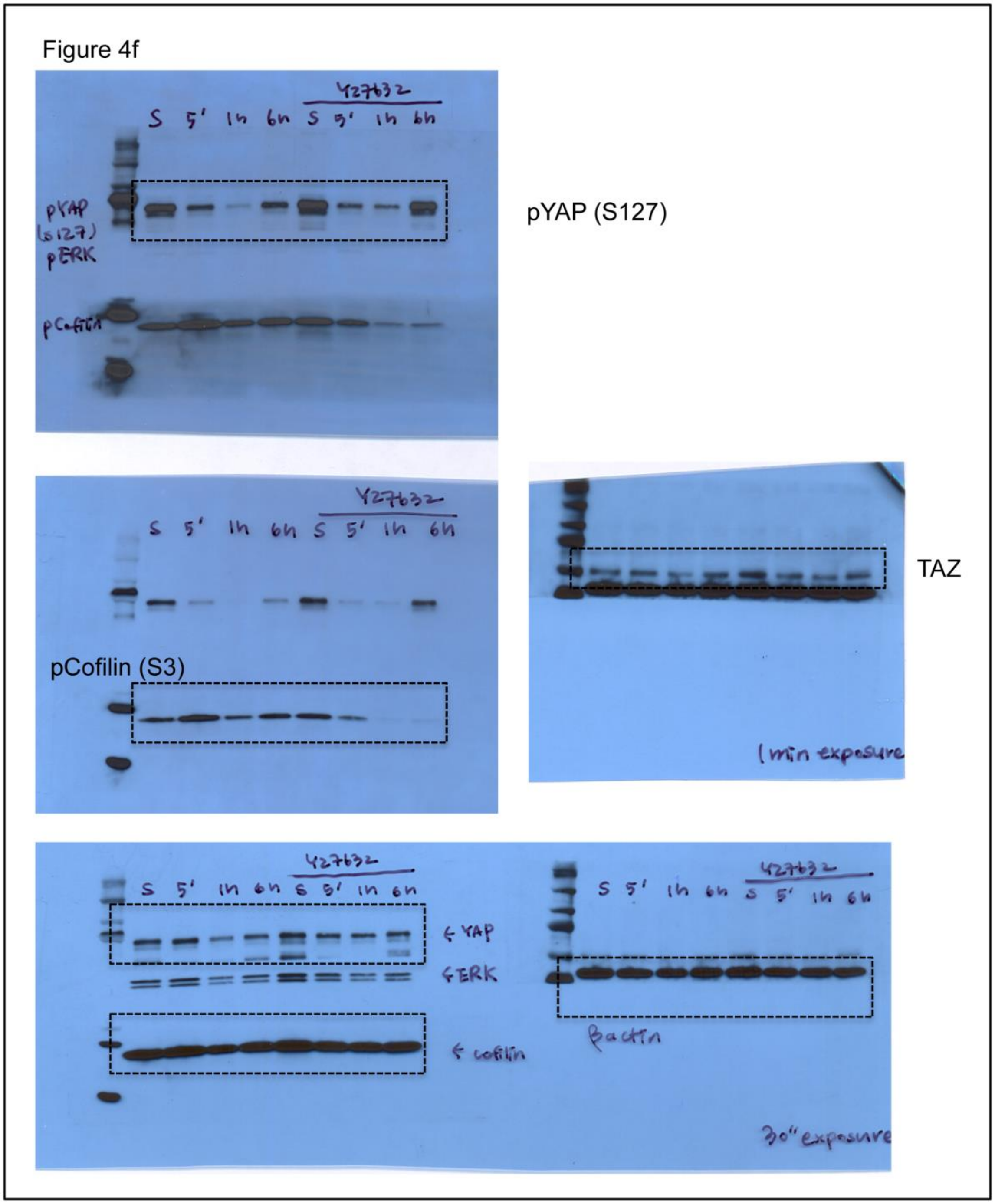

Supplementary Figure 11. Full gel blots (continued)

Figure 4h

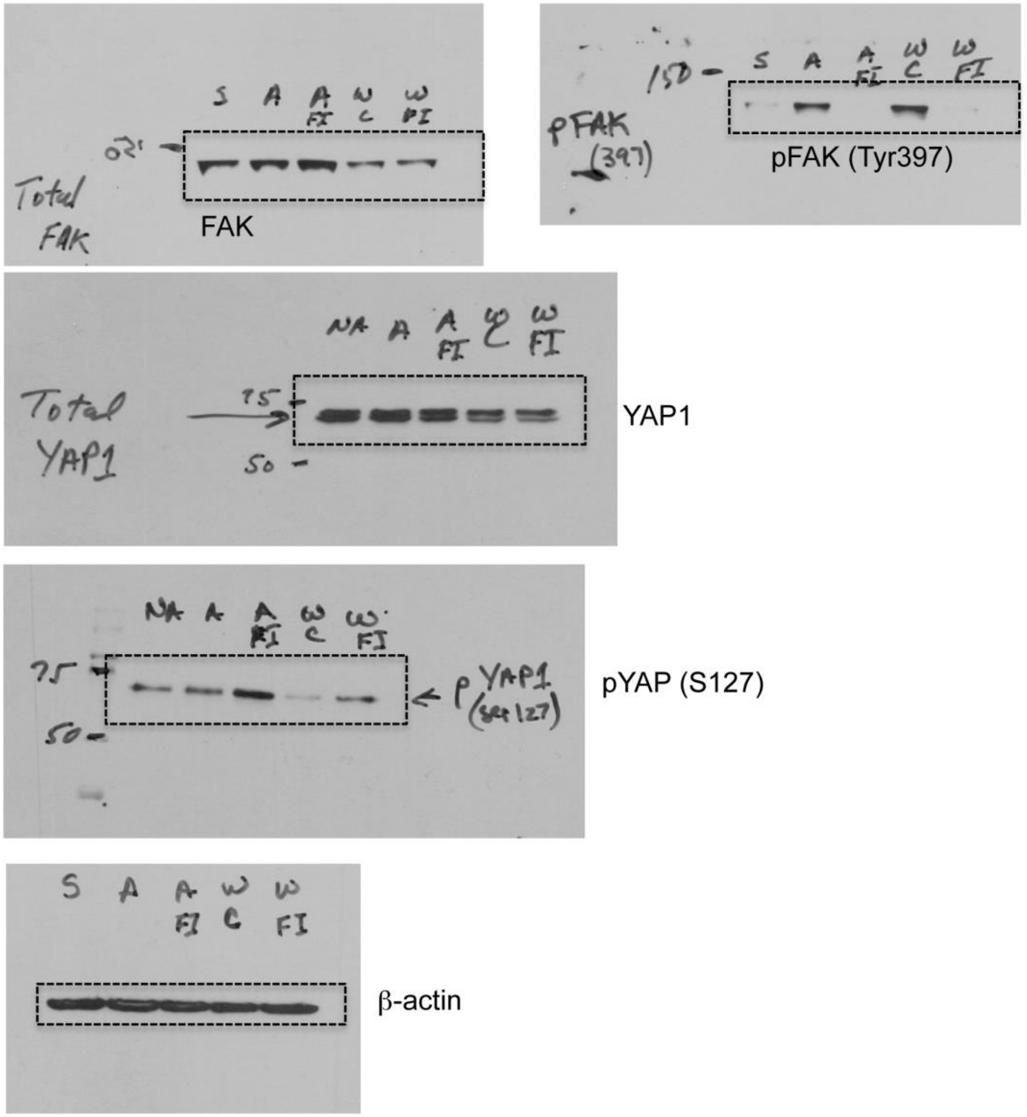

Supplementary Figure 11. Full gel blots (continued)

Figure 5a

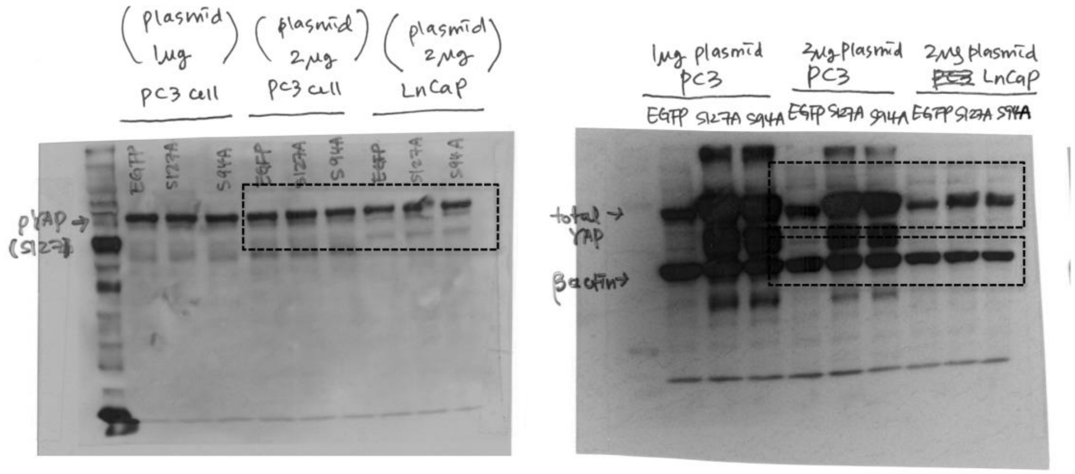

Supplementary Figure 5d

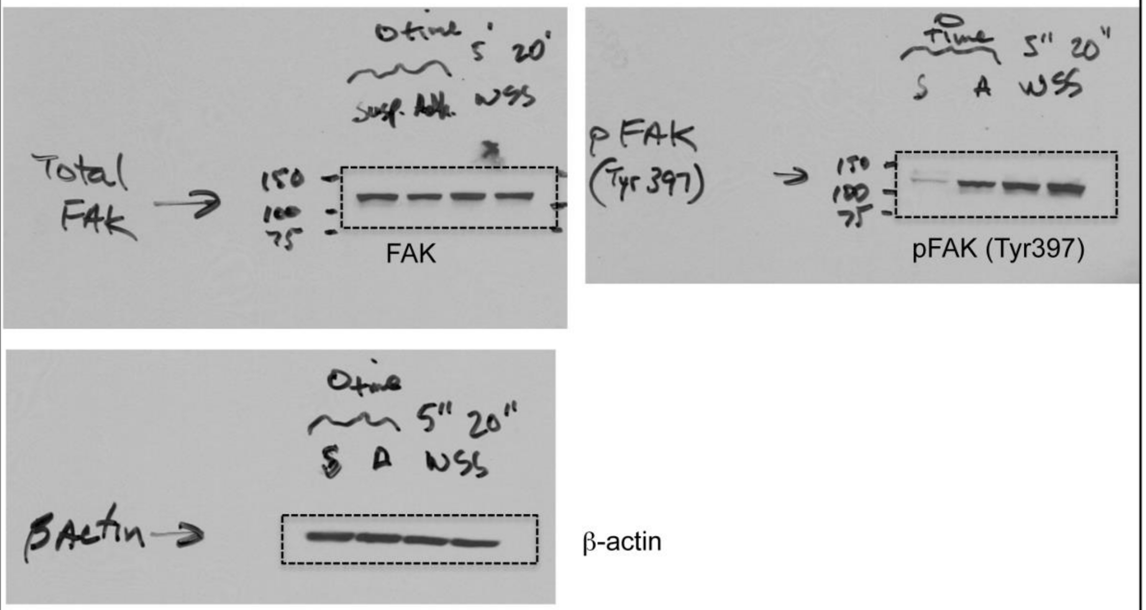

Supplementary Figure 11. Full gel blots (continued)

Supplementary Figure 5e

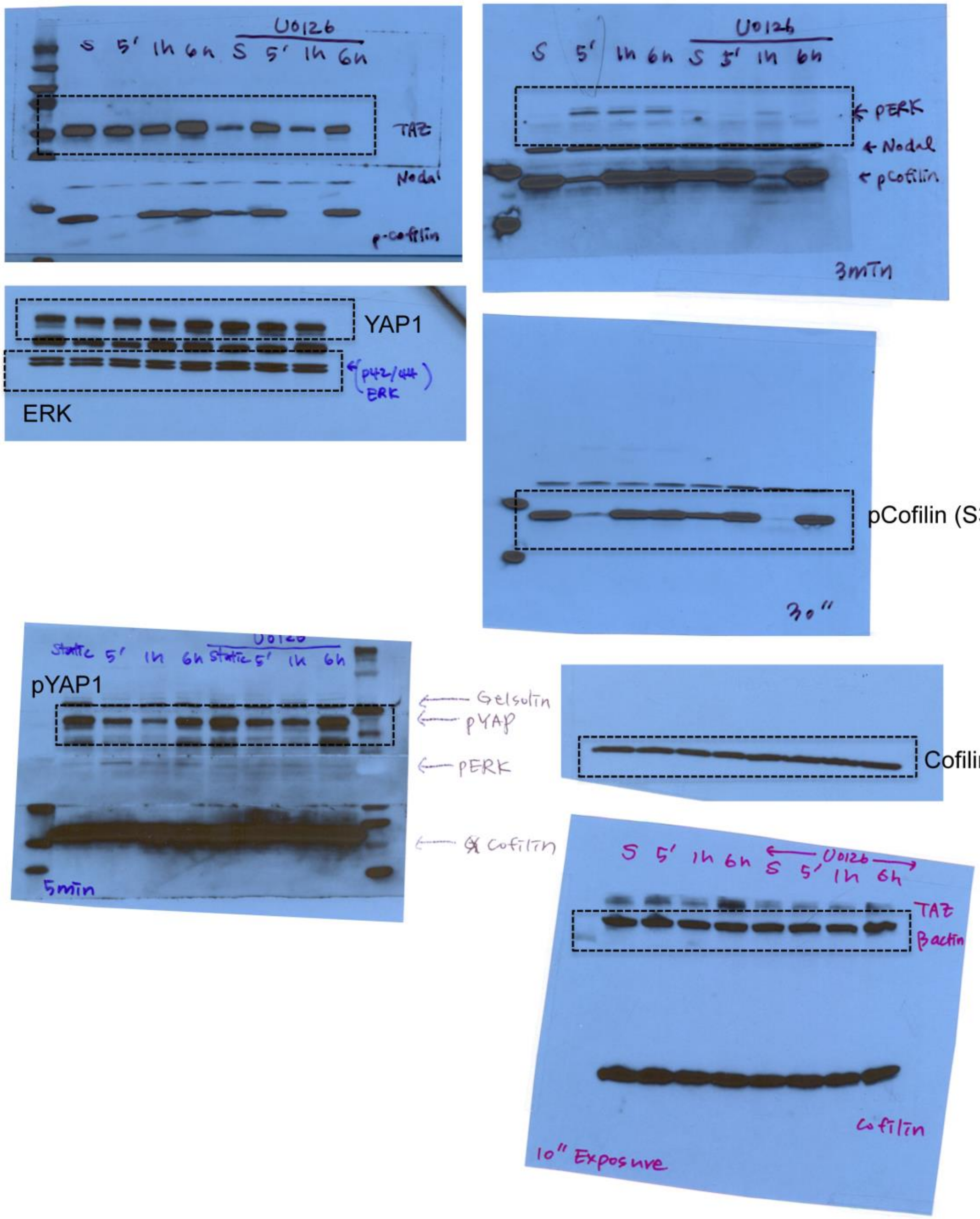

Supplement: Supplementary Information — Supplementary Figures 1-11. [file ncomms14122-s1.pdf]
